# Basal Cell Carcinoma in Gorlin’s Patients: a Matter of Fibroblasts-Led Protumoral Microenvironment?

**DOI:** 10.1371/journal.pone.0145369

**Published:** 2015-12-22

**Authors:** Yannick Gache, Florence Brellier, Sophie Rouanet, Sahar Al-Qaraghuli, Maria Goncalves-Maia, Elodie Burty-Valin, Stéphanie Barnay, Sabine Scarzello, Martial Ruat, Nicolas Sevenet, Marie-Françoise Avril, Thierry Magnaldo

**Affiliations:** 1 INSERM U1081—CNRS UMR7284 –UNS, Nice, France; 2 Université de Nice–Sophia-Antipolis, Faculté de Médecine, Nice, France; 3 CNRS FRE2939, Université de Paris Sud—Institut Gustave Roussy, Villejuif, France; 4 CNRS UMR9197, Neuroscience Paris-Saclay Institute, Gif‑sur‑Yvette, France; 5 INSERM U916 & Institut Bergonié, Laboratoire de génétique moléculaire, Bordeaux, France; 6 Service de Dermatologie, Université Paris 5-APHP, Paris, France; 7 UMR 7138, Université Pierre-et-Marie-Curie, Paris, France; San Gallicano Dermatologic Institute, ITALY

## Abstract

Basal cell carcinoma (BCC) is the commonest tumor in human. About 70% sporadic BCCs bear somatic mutations in the *PATCHED1* tumor suppressor gene which encodes the receptor for the Sonic Hedgehog morphogen (SHH). *PATCHED1* germinal mutations are associated with the dominant Nevoid Basal Cell Carcinoma Syndrome (NBCCS), a major hallmark of which is a high susceptibility to BCCs. Although the vast majority of sporadic BCCs arises exclusively in sun exposed skin areas, 40 to 50% BCCs from NBCCS patients develop in non photo-exposed skin. Since overwhelming evidences indicate that microenvironment may both be modified by- and influence the- epithelial tumor, we hypothesized that NBCCS fibroblasts could contribute to BCCs in NBCCS patients, notably those developing in non photo-exposed skin areas. The functional impact of NBCCS fibroblasts was then assessed in organotypic skin cultures with control keratinocytes. Onset of epidermal differentiation was delayed in the presence of primary NBCCS fibroblasts. Unexpectedly, keratinocyte proliferation was severely reduced and showed high levels of nuclear P53 in both organotypic skin cultures and in fibroblast-led conditioning experiments. However, in spite of increased levels of senescence associated β-galactosidase activity in keratinocytes cultured in the presence of medium conditioned by NBCCS fibroblasts, we failed to observe activation of P16 and P21 and then of bona fide features of senescence. Constitutive extinction of P53 in WT keratinocytes resulted in an invasive phenotype in the presence of NBCCS fibroblasts. Finally, we found that expression of SHH was limited to fibroblasts but was dependent on the presence of keratinocytes. Inhibition of SHH binding resulted in improved epidermal morphogenesis. Altogether, these data suggest that the repertoire of diffusible factors (including SHH) expressed by primary NBCCS fibroblasts generate a stress affecting keratinocytes behavior and epidermal homeostasis. Our findings suggest that defects in dermo/epidermal interactions could contribute to BCC susceptibility in NBCCS patients.

## Introduction

In the human, most sporadic cancers are from epithelial origin and are thought to arise following sequences of genotoxic injuries [1]. The vast majority of cancers affecting the general population (about 30%) derive from skin, notably from the epidermal compartment. The ultraviolet (UV) content of the sunlight is by far the most frequent etiological factor in cutaneous carcinogenesis; in this context, exposure to sunlight is responsible for basal- (BCC), and squamous- cell carcinoma (SCC) as well as malignant melanoma (MM) [2]. Patients suffering from the dominantly inherited disease Nevoid Basal Cell Carcinoma/Gorlin-Goltz Syndrome (NBCCS) [3] express a variety of traits including developmental failures such as polysyndactily, bifid ribs, odontogenic keratocysts, as well as proneness toward medulloblastoma and ovarian cancers. However, the most severe genetic affliction in NBCCS patients is by far their constitutive susceptibility toward BCCs [3, 4, 5]. For yet unexplained reasons, 40 to 50% of those NBCCS BCCs, develop in non photo-exposed skin areas, [6] suggesting that susceptibility toward BCCs is not linked to inappropriate responses to DNA damages such as observed in the xeroderma pigmentosum nucleotide excision repair genetic syndrome [7].

Germinal mutations responsible for the NBCCS encompass the *PATCHED1* (*PTCH1*) tumor suppressor gene, most of which are non-sense and are thought to be deleterious. No evidence of a clear genotype-phenotype relationship has yet been put forward. PATCHED is a putative 12-pass trans-membrane receptor that binds the diffusible morphogen molecule SONIC HEDGEHOG (SHH) [8–10]. In brief, control of the pathway depends on the absence or the presence of SHH. In the absence of SHH, PATCHED acts as a repressor leading to inactivation of the GLI transcriptional activators. Upon receipt of the SHH signal, active forms of GLI transcriptional factors are produced to modulate the expression of SHH target genes [11].

In more than 50% sporadic BCCs, somatic mutations in the *PATCHED1* gene have also been identified, with frequent loss of heterozygosity including the *PATCHED1* locus [12]. However, since virtually 100% sporadic BCCs exhibited accumulation of *GLI* mRNAs, [13] it has been proposed that constitutive de-repression of the SHH/PATCHED pathway could result in inappropriate control of the cell proliferation and differentiation balance, developmental failures and cancer development, most notably BCCs [11].

In sporadic cancers, overwhelming evidences have accumulated indicating that the microenvironment of epithelial cells could play a decisive role in the onset, growth, and fate of neoplastic cells. Cancer cells can activate their microenvironment, leading to remodeling of components of the extracellular matrix and tumor cell growth through *de novo* secretion of various cytokines and growth factors [14]. In animal models, the tumor microenvironment has also been shown to generate oxidative damages and genetic instability, hence promoting invasive capacity of epithelial cells [15].

Based on the fact that sun exposure is not an essential etiological factor of NBCCS BCCs, [16] we investigated whether altered dermo-epidermal interactions may play a role in BCCs predisposition of NBCCS patients. More specifically, as the SHH/PATCHED pathway is known to be involved in intercellular control of proliferation, we hypothesized that deregulation of the SHH/PATCHED pathway in mesenchymal cells may affect proper control of epithelial cell growth and contributes to the expression of microenvironmental factors leading to the development of BCCs. In this respect, our previous transcriptome analyses of primary NBCCS fibroblasts cultured in 3D organotypic systems, revealed their significant inclination to express a phenotype closely resembling that of fibroblasts associated to sporadic BCCs [17, 18]. The most significant differential expression between wild type (WT) and NBCCS fibroblasts concerns proteins involved in the composition of the extracellular matrix and its remodeling, cellular proliferation, invasion, angiogenesis, and control of the production of reactive oxygen species (ROS).

In the present study, we aimed at better understanding the fact that NBCCS patients develop BCCs in skin areas preserved from apparent genotoxic insults. We took into account that mesodermal-ectodermal interactions play a major role upon development, maintenance and repair of adult organs as well as cancer development [19]. Our results indicate that the presence of *PATCHED1* +/- NBCCS fibroblasts in the dermal compartment of organotypic skin cultures (OSC) is sufficient for severely perturb epidermal differentiation, proliferation, and cell cycle control in the overlaying WT epidermis. These perturbations, however, did not correlate with expression of *bona fide* markers of apoptosis or senescence. Altogether, the present data strongly suggest for the first time that the dermal microenvironment led by NBCCS fibroblasts may play a functional role in high levels of BCCs in NBCCS patients in absence of external genotoxic stress.

## Materials and Methods

This study was approved by a local French ethic committee (Commité consultatif pour la protection des personnes en recherche biomédicale, CCPPRB: CSET935) and was conducted after informed written consent of NBCCS patients.

### Skin biopsies and primary cell cultures

Human primary fibroblasts and keratinocytes were isolated from healthy, non photo-exposed skin biopsies of either control individuals or NBCCS patients bearing either nonsense or missense mutations in the *PATCHED1* gene (S1 Table).

Primary dermal fibroblasts were grown in DMEM supplemented with 10% fetal calf serum (FCS), 2 mM glutamine, 1% penicillin/streptomycin, 1 mM sodium pyruvate, 1% non essential amino acids, and 0.2% fungizone, at 37°C, 5% CO_2_. Fibroblasts strains (passages P6 to P9) were seeded at 10^4^ cells/cm², and cultured up to 80–90% confluence before processing. Under standard conditions, primary keratinocytes, E6-E7-immortalized keratinocytes, and SCC13 cell line were cultured on feeder layers of lethally irradiated 3T3-J2 Swiss mouse fibroblasts in cFAD keratinocyte medium [20]. When indicated, cFAD was replaced by a FAD medium containing 0.5% FCS. Immortalization of keratinocytes was performed using the pLXSN16E6-E7 retroviral vector [21] and Phoenix helper cells (http://www.stanford.edu/group/nolan/protocols/pro_helper_dep.html).

### Culture of keratinocytes in culture medium conditioned by fibroblasts

Fibroblasts were seeded at a density of 10^4^ cells/cm^2^ and cultured for 72 hours. Fibroblasts were then cultured in FAD containing 0.5% FCS and conditioned supernatants were collected after 48 hours. Keratinocytes were seeded at 11 000 cells/cm² on lethally irradiated 3T3-J2 Swiss mouse fibroblasts in cFAD for 24 hours. Cells were then cultured in FAD containing 0.5% FCS for 24 hours before being incubated in the fibroblasts conditioned media for 72 hours. Keratinocytes were then lysed for RNA and proteins preparations.

### Expression of SHH in culture supernatants from either fibroblasts or fibroblasts and keratinocytes co-cultures

For analyses of SHH secretion by fibroblasts, cells were seeded at 10^4^ cells/cm^2^ and cultured in fibroblasts medium for 48 hours. Cells were then incubated in fibroblasts medium without FCS for 48 hours. For analyses of SHH secretion in fibroblasts-keratinocytes co-cultures, fibroblasts were first seeded at 10^4^ cells/cm^2^ in cFAD for 24 hours. Keratinocytes were then added to the fibroblasts cultures at a density of 7 500 cells/cm^2^_._ Co-cultures of fibroblasts and keratinocytes were incubated in keratinocyte-SFM medium (Gibco, Life technologies, NY, USA) for 48 hours. Conditioned media were then collected, concentrated on Vivaspin columns (Sartorius Stedim Biotech GmbH, Goettingen, Germany), and protein extracts were analysed by western blot. Three independent conditioning experiments were performed.

### Organotypic skin cultures

OSC were prepared as described [22]. Briefly, human dermal fibroblasts (passages P5 to P8) were embedded into a type I bovine collagen gel at a concentration of 2.10^6^ cells/ml. After 4 days of contraction of the dermis equivalent, 5.10^4^ keratinocytes were seeded onto the dermis equivalent and cultured in immersion for 7 days before being raised at the air-liquid interface for 7 additional days. When indicated, OSC were processed in the presence of 5 μg/ml of either the anti-SHH 5E1 monoclonal antibody (mAb) (DHSB, Iowa, IA, USA) or the isotype-matched anti-cMyc 9E10 mAb (Biomol, Plymouth Meeting, PA, USA). OSC were then processed for paraffin inclusion or snap freezing in liquid nitrogen.

### Quantitative invasion assay

Organotypic culture system was set up according to Gaggioli [23]. Briefly, 5.10^5^ fibroblasts were embedded in a mixture of collagen I (#354249; BD Biosciences, Oxford, UK) and Matrigel (#354234; BD Biosciences). After 1 hour at 37°C, 4.10^5^ keratinocytes were plated on top of the gels, and incubated overnight at 37°C, 10% CO2. Gels were then mounted at the air-liquid interface and fed through capillarity diffusion by cFAD culture medium. After 7 days, the cultures were harvested and fixed using 4% paraformaldehyde, 0.25% glutaraldehyde. The invasion index [1 –(non-invading keratinocytes area/invading and non invading cells area)] values represents the average of 3 total sections from two independent organotypic skin cultures.

### Histology and immunostaining

Hematoxylin-Eosin (H&E), immuno labellings on paraffin sections, and indirect immunofluorescence analyses on frozen sections were performed as described [24]. Immunostaining of Laminin B1, β1 Integrin, and P53 were performed on paraffin sections using rabbit anti-Laminin B1 serum (1/400; Novotec, Paris, France); anti-β1 integrin mAb (1/50; clone 4B7, Oncogene Research Products, Boston, MA, USA); P53 monoclonal antibody (1/50; clone DO-7, Dako, Glustrup, Denmark), respectively. Staining was revealed using appropriate secondary antibodies linked to alkaline phosphatase and the Envision kit (Dako, Glustrup, Denmark) followed by H&E counter-staining. Immunostainings of Keratin 10, Loricrin, and Ki67 were performed on air-dried cryosections using the anti-Keratin 10 mouse mAb (1/10; RKSE 60, Sanbio, Uden, The Netherlands), anti-Loricrin rabbit antiserum (1/200, [25]) and Ki67 mAb (1/20, NovoCastra, Newcastle, UK).

### Senescence-associated β-galactosidase (SA-β-Gal) activity

Fibroblasts were seeded at a density of 8 500 cells/cm^2^ in fibroblasts medium for 48 hours. Fibroblasts were then cultured in FAD medium depleted in calcium and containing 0.5% FCS and conditioned supernatants were collected after 72 hours. WT keratinocytes (2 000 cells/cm^2^), SCC13 cells (2 000 cells/cm^2^), and WT E6-E7 keratinocytes (650 cells/cm^2^) were seeded onto glass coverslips in cFAD for 72 hours. Cells were then cultured in calcium-free FAD medium containing 0.5% FCS for 24 hours before being incubated in the fibroblasts conditioned media for 48 hours. SA-β-Gal activity in cultured keratinocytes was detected using the SA-β-Gal staining kit (Cell Signaling Technology, Danvers, MA, USA) according to the manufacturer's recommendations. For positive control of SA-β-Gal activity, WT keratinocytes were treated for 2 hours with 200 μM H2O2. After processing, cells were photographed under reflected light. The ratio of SA-β-Gal positive cells / total cell numbers was determined by blind counting of 10 fields / slide by 3 independent experimentators.

### Protein extraction and western blotting

For western blot detection of P53, P16, and P21, proteins were solubilized in 8 M Urea lysis buffer (8 M Urea, 50 mM Tris-HCl, 0.1 M β-Mercaptoethanol, 1 mM DTT, 100 μg/ml phenylmethanesulfonylfluoride). Proteins (15 μg) were separated by 8% (P53) or 14% (P21, P16) SDS-PAGE, blotted onto polyvinyl difluoride (PVDF) membrane (Millipore, Corporation, Billerica, MA, USA), and revealed using the anti-P53 DO-7 mAb (1/1000; Dako, Glustrup, Denmark), the anti-P21 EA10 mAb (0.5 μg/ml; Abcam ab16767, Cambridge, UK), or the anti-human-P16 mAb (1/1 000, BD Biosciences, Le Pont de Claix, France). Membranes were re probed using the anti-GAPDH mAb (1/2000; Abcam 9484, Cambridge, UK) or the anti-β-Tubulin TUB2.1 mAb (1/5 000, Sigma Aldrich, St Louis, MO) for loading/transfer controls. Membranes were then analysed using the Luminata crescendo western HRP substrate (Millipore Corporation, Billerica, MA, USA). Quantification of P53 was performed using the ImageJ software. Quantification of P21 and P16 was performed using the Fusion-Capt software (Vilber Lourmat, Eberhardzell, Germany). For detection of SHH, 1μg of concentrated culture supernatants were subjected to 10% SDS-PAGE, blotted, and revealed using the N-SHH 167Ab polyclonal antibody [26]. Quantification of N-SHH was performed using GeneGnome device and GeneTools software (Syngene, Synoptics Ltd, Cambridge, UK).

### RNA extraction and quantitative RT-PCR

Total RNA was extracted using the RNeasy Mini kit (Qiagen, Hilden, Germany). Reverse transcription was performed from 1 μg RNA using the Superscript II Reverse Transcriptase (Roche Applied Science, Basel, Switzerland) and random primers. Real Time-PCR was carried out on cDNAs using primers listed in S2 Table and the 7900 HT Fast Real-Time PCR System (Applied Biosystems, Foster City, CA, USA). Results were normalized using the GeNorm software (http://medgen.ugent.be/Bjvdesomp/genorm/). Statistical analysis was performed using the Mann-Whitney test. Semi-quantitative PCR were performed using *PATCHED1* primers F: 5′-GATAAGAGCTCCGGGGGATTC-3′ and R: 5′-CACAGTAGCTTAGGCTTCAGCCC-3′, and *GAPDH* primers F: 5’-CCAAGGCTGTGGGCAAGGTCAT-3’ and R: 5’-TGACAAGGTGCGGCTCCCTAGG-3’ as described [27]. PCR products were quantified after electrophoretic separation in 2% agarose gel, and SYBR fluorescence scanning (STORM; Molecular Dynamics, Sunnyvale, CA) using the ImageQuant software (Amersham Biosciences, Amersham, United Kingdom).

### Transcriptional activity of the *PATCHED1* gene promoter

Keratinocytes were seeded at a density of 16 000 cells/cm^2^. Transient transfections were performed as described [27]. The RSV-β-Gal plasmid was used as a control to normalize transfection efficiency. After transfection, keratinocytes were maintained for 72 hours in medium conditioned from control or NBCCS fibroblasts and the luciferase and β-Gal enzymes activities were measured using the luciferase assay system and β-Gal enzyme assay system kits (Promega Corporation, Madison, WI, USA) [27].

### Reactive oxygen species (ROS)

Accumulation of ROS was examined by using 2’,7’-dichloro-fluorescin diacetate (CM-DFHDA) (Invitrogen^TM^/Molecular probes, Eugene, Oregon, USA). Keratinocytes were seeded at 11 000 cells/cm^2^ in cFAD for 48 hours. Cells were then incubated in fibroblast-conditioned media for 1 hour and then incubated in CM-DCFHDA in HBSS for 30 min at 37°C. Cells were then washed, dissociated, and resuspended in HBSS. Fluorescence was recorded at an excitation/emission wavelength of 485 nm/530 nm by flow cytometry on a FACS Calibur flow cytometer (BD Biosciences, San Jose, CA, USA).

### Statistical analyses

For the box plot representation, the line in the middle of the box represents the median. The box extends from the 25^th^ percentile to the 75^th^ percentile. The lines emerging from the box extend to the upper and lower adjacent values. Significance of the differences between experimental values measured in NBCCS and in WT fibroblasts was assessed using the Mann-Whitney test.

## Results

### NBCCS fibroblasts impact homeostasis of wild type epidermis

**Fig 1:**
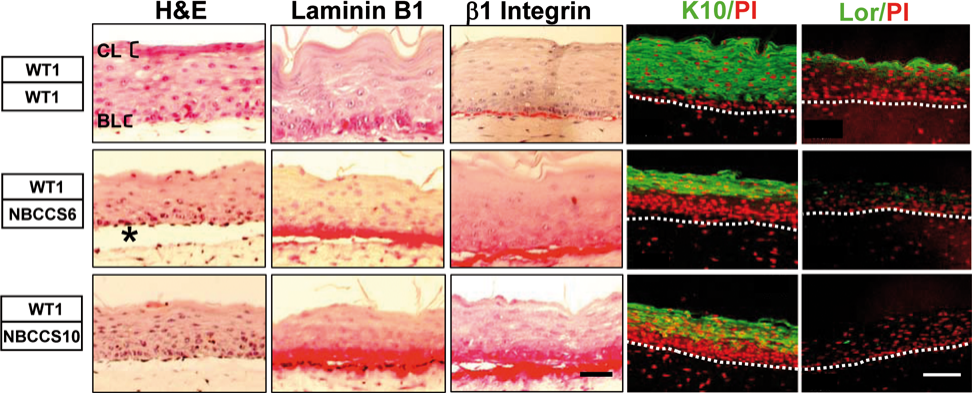
NBCCS fibroblasts alter morphology and differentiation of WT epidermis. WT keratinocytes were seeded onto dermis equivalents comprising skin fibroblasts isolated from either a WT (WT1) donor or from two independent NBCCS patients (NBCCS6 and NBCCS10). H&E staining reveals the absence of cornified layers (CL) and slight epidermal thinning in NBCCS OSC. Note increased accumulation of Laminin B1 and β1 Integrin in the basal layer (BL) and the para basal layers of epidermis. Star (*) points dermo epidermal cleft between the dermal and the epidermal compartments. Also note delayed expression of Keratin 10 (K10) and absence of expression of Loricrin (Lor) in NBCCS OSC; PI, nuclei counter staining using propidium iodide. Dash lines delineate the dermo epidermal junction. Bar: 110 μm

We first analyzed the potential impact of NBCCS primary fibroblasts on epidermal morphogenesis using OSC. NBCCS fibroblasts from two independent patients bearing distinct *PATCHED1* nonsense mutations (NBCCS6: c.1925dupC; p.Pro643ThrfsX11 and NBCCS10: c.1366delA; p.Thr456ProfsX35) were incorporated in a dermis equivalent (*PATCHED* + /-) overlaid with a stratified squamous epithelium developed from primary WT keratinocytes (*PATCHED* +/+) (NBCCS OSC) and compared to fully WT OSC. Under these circumstances, the presence of NBCCS fibroblasts in the dermis equivalent was accompanied by a decrease in the thickness of the epidermis, the absence of *bona fide* cornified layers and the persistence of nuclei in the upper suprabasal epidermal layers (parakeratosis) (Fig 1). In addition, the presence of NBCCS fibroblasts was accompanied by clefts at the dermo-epidermal junction, a feature of human BCCs [28]. In contrast, fully WT OSC showed *bona fide* cornified layers and the absence of such parakeratotic features. Immunolabelling of OSC containing NBCCS fibroblasts indicated that deposition of Laminin B1, a major component of the basement membrane, and of β1 Integrin, a trans-membrane protein essential for the attachment of basal keratinocytes to the basement membrane, were increased compared to WT OSC. In addition, β1 Integrin, which is normally limited to keratinocytes located in the basal layer, extended in the first supra basal cell layers. To confirm the relevance of these results, skin sections from patients NBCCS6 and NBCCS10 were also submitted to histological analyses and labelled using anti-β1 Integrin and anti-Laminin B1 antibodies. Although no gross histological alteration was observed, the apparent amount of β1 Integrin and Laminin B1 was found to be either slightly or more strongly increased in NBCCS, respectively, compared to control skin (S1 Fig).

In NBCCS OSC, labelling of the Keratin 10 (K10), a differentiation marker whose normal onset of expression occurs in parabasal, post-mitotic epidermal cell layers, was extended to the second to third rows of epidermal cells (Fig 1). Finally, Loricrin, a precursor of cornified envelopes in terminally differentiated keratinocytes, [25] became barely detectable or even absent in the presence of NBCCS fibroblasts.

### NBCCS fibroblasts provoke stabilisation of P53 in WT keratinocytes

**Fig 2:**
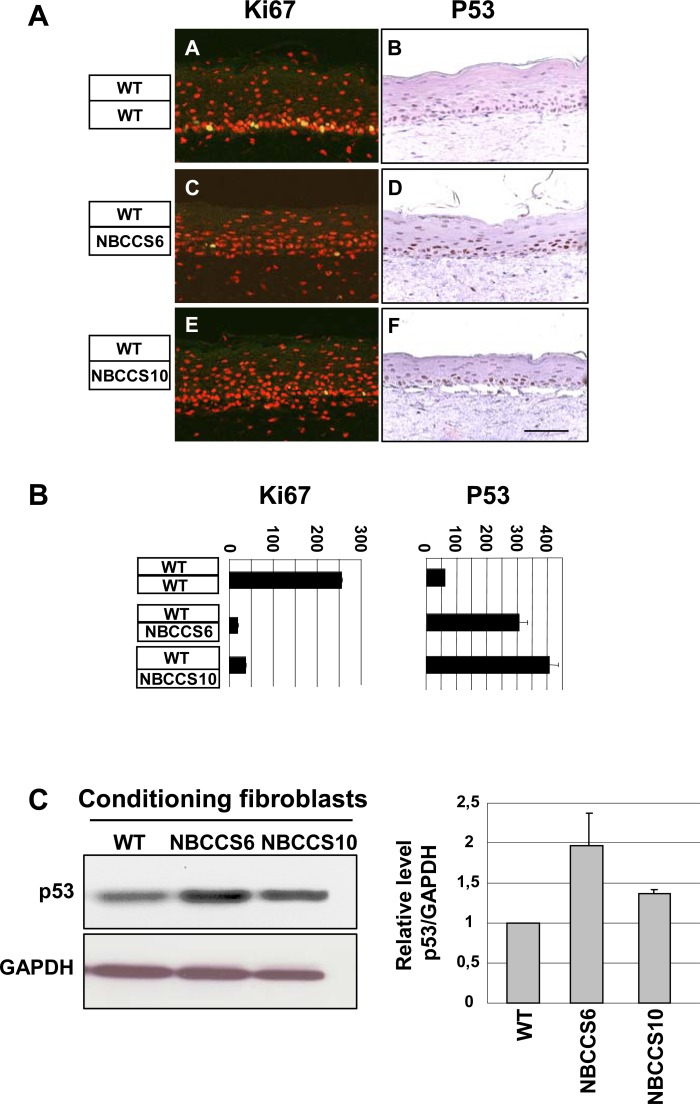
NBCCS fibroblasts alter proliferation of WT keratinocytes in organotypic skin cultures. (A) Rates of cycling keratinocyte (Ki67, green/yellow nuclei), and P53-positive keratinocytes (P53, brown nuclei) were blind counted by 3 independent investigators on sections of OSCs, as indicated. Bar: 170 μm. (B) Ki67- (left panel) and P53- (right panel) positive keratinocytes were counted in the basal layer of 10 different fields spread along 3 independent sections. Error bars indicate the mean ± SD of the counted fields. (C), Western blot analysis shows stabilization of P53 in WT keratinocytes (WT1) treated for 24 h in culture supernatants conditioned by NBCCS (NBCCS6 and NBCCS10) fibroblasts (left panel). Right panel shows levels of P53 relative to GAPDH and expressed as fold induction relative to the WT fibroblasts strain. Error bars indicate the mean ± SD of triplicate experiments.

Taken the above data into account, we aimed at measuring the number of cycling epidermal-keratinocytes positive for Ki-67 labelling in the presence of NBCCS fibroblasts within dermis equivalents. Surprisingly however, number of Ki-67 positive keratinocytes was about 10 times lower in the presence of NBCCS fibroblasts than in the presence of WT fibroblasts (Fig 2). To explore the possible mechanism(s) responsible for the decreased proliferation of WT epidermal keratinocytes located in the vicinity of NBCCS fibroblasts, we analyzed the expression of the P53 tumor suppressor protein provided its function as a major effector of cell cycle arrest at G1-S and G2-M check points [29].

In NBCCS OSC, the number of P53-positive nuclei was enhanced 5.5 to 7.5 times (Fig 2A and 2B) as compared to WT OSC. These observations indicated that the presence of NBCCS fibroblasts within the dermis equivalent was sufficient to severely perturb keratinocyte proliferation as well as the early and subsequent steps of epidermal differentiation. Further, due to the 3D architecture of OSCs, these results suggested to us that NBCCS fibroblasts could perturb skin homeostasis through diffusible molecules accommodating a dialog between the dermis and the epidermis.

### How NBCCS fibroblasts may alter behavior of epidermal keratinocytes?

Given that NBCCS fibroblasts could impact epidermal homeostasis through diffusible molecules, we assessed in 2D culture conditions expression of secreted proteins as previously reported in NBCCS fibroblasts cultured in 3D dermis equivalents in the absence of epidermal keratinocytes [17]. Under these circumstances, a minimal fraction of 9 of the 18 mRNA signature was differentially expressed (significantly) in WT versus NBCCS fibroblasts (Table 1). Among these mRNAs, the average levels of the anti-angiogenic proteins angiopoietin-like 4 (*ANGPTL4*) and matrix GLA protein (*MGP*) mRNAs were increased by 5.6 (p<0.025) and 61.7 (p<0.05), respectively, in NBCCS compared to WT cells. As well, relative levels of the C-X-C ligand 12 (*CXCL12*) and fibroblast growth factor 7 (*FGF7*) mRNAs were increased in NBCCS fibroblasts by 4.0 and 2.9 fold, respectively (p<0.025). The WNT/-catenin signaling pathway is known to be activated in CAFs [30, 31]. In 2D cultures of NBCCS fibroblasts, 4 mRNAs of the WNT/-catenin pathway were found mis-regulated as in 3D dermis equivalents. Amount of mRNAs encoding *WNT5A* (ligand) and Dickkopf 3 (*DKK3*) (inhibitor) were decreased by 5.0 and 2.0 (p<0.025), respectively. Amount of mRNAs of the WNT target genes *WISP2* (WNT1 inducible signaling pathway protein 2) and *ID2* (inhibitor of DNA binding 2) were increased by 4.9 (p<0.05) and 3.2 (p<0.025), respectively. As in 3D conditions, levels of *COL7A1* mRNA were decreased in NBCCS fibroblasts cultured in 2D conditions compared to control cells (5.0 fold; p<0.05). In contrast, mRNAs encoding MMPs (1 and 3), COL11A1, COL3A1, LAMA2, TNC, GREM1, ANGPTL2, and SFRP2 were no longer mis-regulated in NBCCS fibroblasts cultured in 2D conditions while they were in 3D. These results indicated that a fraction of transcriptional deregulations previously described in 3D NBCCS dermis equivalents are conserved under 2D culture conditions, hence allowing fibroblast-led conditioning experiments of epidermal keratinocytes.

**Table 1:** Average mRNA expression levels in WT or NBCCS fibroblasts in either 2D or 3D cultures conditions.

Genes Name	2D	3D		
Average NBCCS / average WT	NBCCS <or> WT p-value	Average NBCCS / average WT	NBCCS <or> WT p-value	
*MMP1*	12.1	NS	8.4	p<0.025
*MMP3*	4.1	NS	26.8	p<0.025
*COL3A1*	0.8	NS	0.7	p<0.05
*COL7A1*	0.3	p<0.025	0.6	p<0.05
*COL11A1*	7.2	NS	11.5	NS
*LAMA2*	1.0	NS	0.4	p<0.025
*TNC*	0.5	NS	1.2	NS
*CXCL12*	4.0	p<0.025	2.4	p<0.025
*MGP*	61.7	p<0.05	5.2	p<0.025
*ANGPTL2*	1.7	NS	3.0	p<0.025
*ANGPTL4*	5.6	p<0.025	9.8	p<0.025
*FGF7*	2.9	p<0.025	2.3	p<0.05
*GREM1*	1.6	NS	1.7	NS
*SFRP2*	0.7	NS	3.5	p<0.025
*DKK3*	0.5	p<0.025	0.3	p<0.025
*WNT5A*	0.2	p<0.025	0.5	p<0.05
*WISP2*	4.9	p<0.05	2.7	p<0.05
*ID2*	3.2	p<0.025	2.6	p<0.025

The level of mRNA of the indicated genes was measured by RT-qPCR on the mRNAs extracted from cultures of fibroblasts in 2D conditions or from dermal equivalents (3D) (WT: n = 3; NBCCS: n = 6). The ratio between the average mRNA levels in the NBCCS and the WT fibroblasts and the p-value are indicated. NS: not significant (p>0.05).

### NBCCS fibroblasts alter keratinocytes behavior through diffusible molecules

Whether diffusible factors expressed by NBCCS fibroblasts could act on stabilization of P53 in epidermal cells was assessed by treating WT keratinocytes with culture supernatants conditioned by either WT or NBCCS fibroblasts. Western blot analyses showed that levels of P53 were slightly increased in WT keratinocytes treated with culture supernatants of NBCCS fibroblasts (Fig 2C). These observations strongly suggested that NBCCS fibroblasts secrete molecules leading to P53 stabilization and cell cycle modifications in WT keratinocytes. Among the identified factors showing altered expression in NBCCS fibroblasts, ANGPTL4 has been shown to elevate the intracellular O2:H202 ratio in a manner dependent of β-1 and β-4 Integrins and to confer anoikis resistance to tumors cells [32]. Based on these observations, we assessed whether overexpression of ANGPL4 in NBCCS fibroblasts could provoke a paracrine stress in epidermal keratinocytes, hence leading to P53 stabilization. However, quantifications of reactive oxygen species (ROS) in WT keratinocytes conditioned in either primary WT or NBCCS fibroblasts culture supernatants, failed to reveal any difference among our experimental settings (S2 Fig). Finally, in spite of a slight increase of P53 stabilization in WT keratinocytes cultured in medium conditioned by WT or NBCCS fibroblasts, we failed to detect any significant variation in cell cycle features (not shown). Consistently, the check point protein P21, a canonical target of P53, was not increased (but even rather slightly decreased) under our experimental conditions, suggesting that stabilization of P53 in 2-D cultures was not sufficient to provoke significant cell cycle alterations, such as accumulation of keratinocytes in G1 or sub-G1 phases.

**Fig 3:**
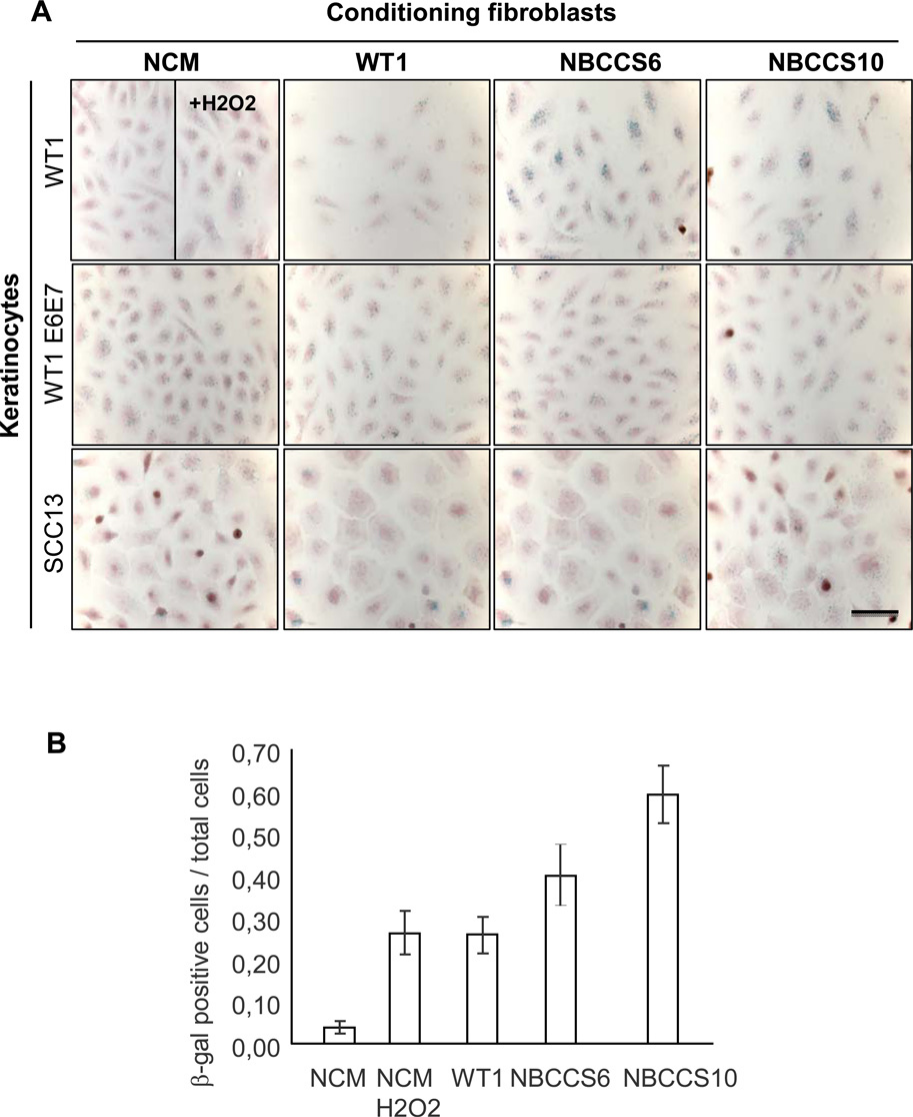
Culture supernatants from NBCCS fibroblasts promote SA-β-Gal activity in WT but not in transformed (E6-E7) or tumoral (SCC13) keratinocytes. (A) SA-β-Gal activity in WT1, WT1 E6-E7, and SCC13 keratinocytes after treatment with either non conditioned medium (NCM), culture supernatant conditioned by WT (WT1), or NBCCS (NBCCS6 and NBCCS10) fibroblasts. (B) SA-β-Gal positive cells were blind counted by three independent investigators. Bars represent the confidence intervals (α = 0.05). Number of SA-β-Gal positive cells was increased in WT keratinocytes treated with NBCCS supernatants. No increase of SA-β-Gal positive cells was detected in both WT1 E6-E7 and SCC13 keratinocytes.

Then we hypothesized that NBCCS fibroblasts could alter the behavior of epidermal keratinocytes through a senescent-like mechanism. We measured the accumulation of SA-β-Gal) in keratinocytes with different genetic backgrounds treated with culture supernatants conditioned by WT or NBCCS fibroblasts (Fig 3). Culture supernatants conditioned by NBCCS6 and NBCCS10 fibroblasts induced an increase in the number of SA-β-Gal positive cells in WT1 keratinocytes (1.55-fold and 2.29-fold increases, respectively). In contrast, we did not observe any increase of SA-β-Gal in keratinocytes with P53 altered genetic backgrounds, i.e. in both WT1 E6-E7 or SCC13 keratinocytes.

On the base of the increase of SA-β-Gal in WT keratinocytes conditioned in culture supernatants of NBCCS fibroblasts, we next investigated the role of P21 and P16, two cell-cycle check point regulators that expressions have been described in senescent cells [33]. Surprisingly, however, western blots indicated a marked decrease of P21 and P16 proteins in WT keratinocytes cultured in media conditioned by NBCCS fibroblasts (S3 Fig). These results suggest that the increase of the SA-β-Gal activity described above is not correlated with stabilization of the P21- and P16 check point proteins.

### NBCCS fibroblasts impact the SHH/PATCHED pathway in WT keratinocytes

**Fig 4:**
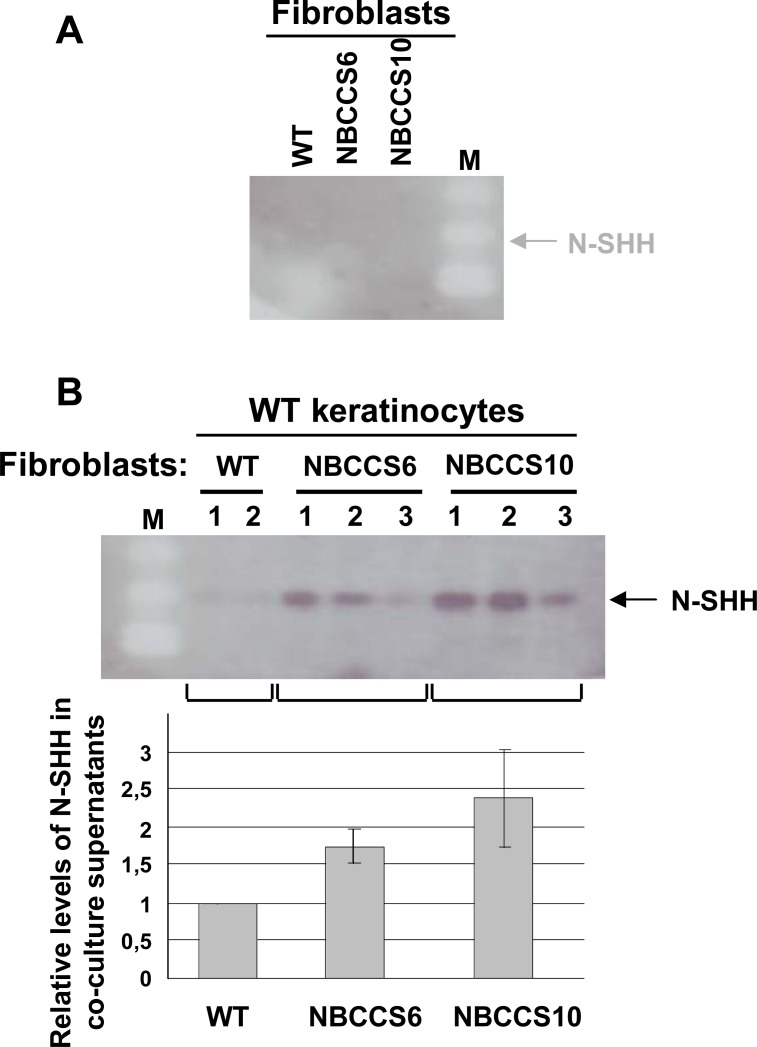
SHH secretion depends on the presence of both fibroblasts and keratinocytes and is further increased in the presence of NBCCS fibroblasts. (A), Western blot analysis of N-SHH secreted from either WT (WT1) or NBCCS (NBCCS6 or NBCCS10) fibroblasts. Secretion of N-SHH was not detectable in culture supernatants of WT and NBCCS fibroblasts. (B), Western blot analysis of N-SHH secreted from co-cultures of WT keratinocytes with WT (WT1) or NBCCS fibroblasts (NBCCS6 or NBCCS10). N-SHH became slightly detectable in the presence of WT fibroblasts (WT1). N-SHH amount was substantially increased in the presence of NBCCS fibroblasts (NBCCS6, NBCC10). Lower panel: quantitation of N-SHH in culture supernatant obtained under the circumstances described for the upper panel. Note that detection of SHH is limited to the N-SHH processed protein (22 kDa, arrow).

Numerous researches have shown that SHH production could be misregulated in various cancer types, notably those involving alteration of mesenchyme / stromal cells [34]. Expression of SHH was thus analyzed in NBCCS and WT fibroblasts and in keratinocytes cultured in 2D. RT-qPCR analysis indicated the absence of SHH mRNA in keratinocytes and hardly detectable expression in fibroblasts (not shown). In neither WT nor NBCCS fibroblast culture supernatants, the active N-terminal fragment of SHH (N-SHH), could be detected by western blot analyses (Fig 4). In contrast, N-SHH became detectable in culture media obtained after 48 hours of co-cultures of WT keratinocytes with WT fibroblasts (Fig 4B). Secretion of N-SHH was further increased in co-cultures of WT keratinocytes with NBCCS fibroblasts (from x 1.8 to x 2.4).

**Fig 5:**
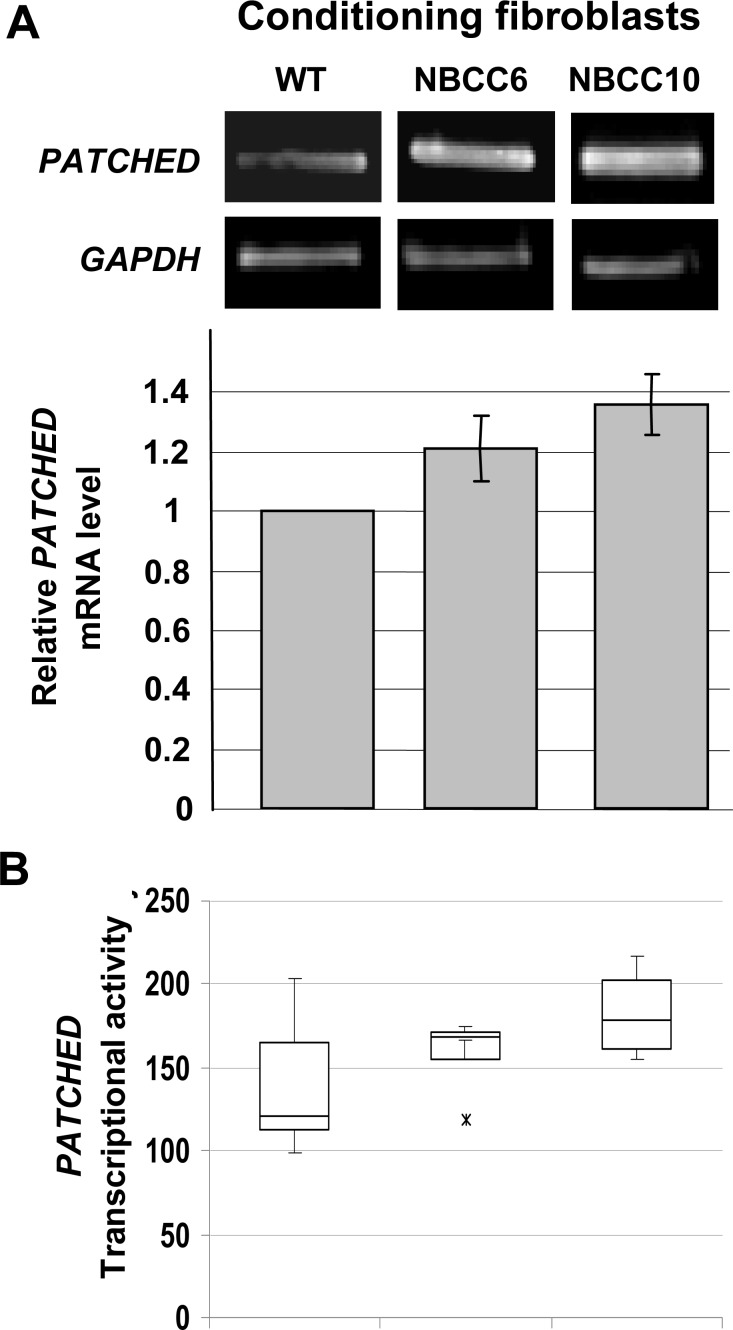
NBCCS fibroblasts stimulate *PATCHED1* transcription in WT keratinocytes. (A), *PATCHED1* and *GAPDH* mRNAs amounts expressed by WT keratinocytes cultured with either WT or NBCCS fibroblasts (NBCCS6 or NBCCS10) were determined after semi-quantitative PCR and agarose gel electrophoresis (upper panel). Relative *PATCHED1* mRNA levels were increased in WT keratinocytes (WT1) treated with culture media derived from NBCCS fibroblasts (NBCCS6 and NBCCS10) (lower panel). (B), Activity of the *PATCHED1* promoter in WT keratinocytes cultured in media conditioned from either WT or NBCCS fibroblasts (NBCCS6 or NBCCS10). Note the substantial increase of the relative transcriptional activity of the *PATCHED1* promoter in WT keratinocytes treated with culture media conditioned by NBCCS fibroblasts. Experiments were performed two times in triplicates, p<0.09.

Since *PATCHED1* is a transcriptional target of the SHH/PATCHED pathway, WT keratinocytes were then conditioned with culture supernatants of NBCCS fibroblasts and levels of *PATCHED1* mRNA in keratinocytes were measured by RT-qPCR. In parallel, WT keratinocytes were transfected with a vector bearing the 4,4 kb 5’ regulatory region of the human *PATCHED1* gene upstream the firefly luciferase reporter gene [27] and cultured in the medium conditioned by NBCCS fibroblasts. Fig 5 indicates that both approaches resulted in a 20–30% increase of *PATCHED1* mRNA amount and transcription. These observation were relevant of a modest but reproducible activation of the SHH pathway in WT keratinocytes exposed to culture supernatants conditioned by NBCCS fibroblasts.

**Fig 6:**
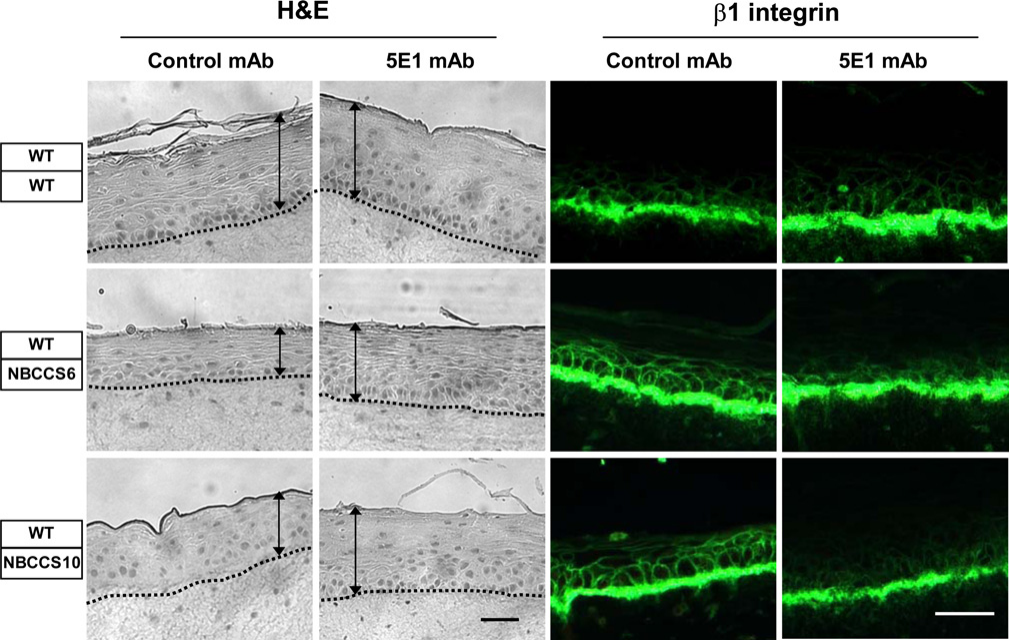
Neutralizing SHH in organotypic skin cultures attenuates alterations of WT epidermis in the presence of NBCCS fibroblasts. OSC comprising either WT (WT1) or NBCCS fibroblasts (NBCCS6 or NBCCS10) were developed in the presence of either the 5E1 anti-SHH blocking mAb (5E1 mAb) or an isotype-matched (anti-Myc 9E10) control antibody (Control mAb). OSC sections were stained with either H&E, or immunolabeled with the anti-β1 Integrin mAb. Note that developing OSC in the presence of the 5E1 antibody improved epidermal atrophy (as pointed out by black arrows) and β1 Integrin delocalization otherwise observed in the presence of NBCCS fibroblasts. Bar: 110 μm

To assess whether SHH secretion by NBCCS dermal fibroblasts could be implicated in epidermal alterations, OSC were developed in the absence or in the presence of the 5E1 anti-SHH blocking antibody. Fig 6 shows that treatment of OSC with the 5E1 blocking antibody (but not an isotype-matched monoclonal antibody) minimized epidermal perturbations observed in the presence of NBCCS fibroblasts. H&E staining revealed increased thickness of the epidermis in NBCCS OSC treated with the blocking 5E1 antibody. Furthermore, labelling of β1 Integrin was normalized to the basal cell layer in the presence of the blocking 5E1 antibody. These data support our observations suggesting that the presence of NBCCS fibroblasts leads to a misactivation of the SHH/PATCHED pathway in the epidermal compartment, and hence, to severe alterations of the behaviour of epidermal keratinocytes.

### NBCCS fibroblasts confer invasiveness of keratinocytes with abrogated P53

**Fig 7:**
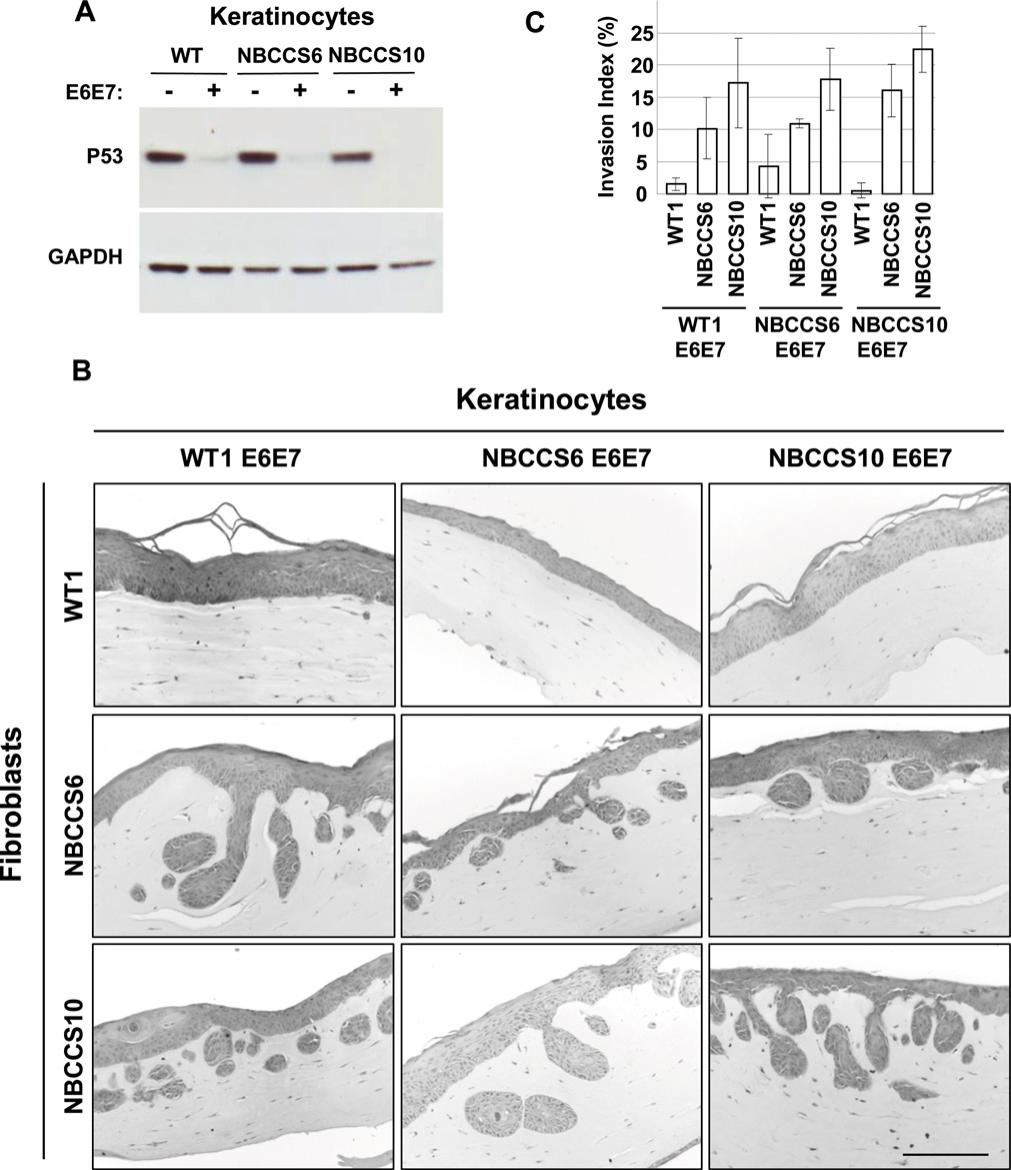
Abrogation of P53 in NBCCS keratinocytes confers invasive properties in the presence of NBCCS fibroblasts. (A) Western blot analysis of P53 showed a strong attenuation in WT (WT1), NBCCS6 and NBCCS10 keratinocytes transduced with a retrovirus expressing the HPV16 E6 and E7 oncoproteins. (B) Representative images of H&E coloration of paraffin-embedded sections of organotypic invasion assays composed of either WT E6-E7 or NBCCS E6-E7 keratinocytes overlaying a dermis equivalent comprising either WT or NBCCS fibroblasts. Bar: 200 μm. (C) Invasion index of keratinocytes from assays as in (B). Values represent the average of 3 total sections from two independent organotypic cultures. Bars represent the confidence intervals, α = 0.05.

Although our data showed that P53 is stabilized in keratinocytes in OSC comprising a NBCCS dermis equivalent or after their incubation in culture supernatants conditioned by NBCCS fibroblasts, we failed to identify the exact nature of such a stress. However, since most skin tumors harbor P53 deleterious mutations (IARC TP53 database, R17 November 2013, www.p53.iarc.fr), [35] we aimed at measuring the impact of P53 abrogation in WT and NBCCS keratinocytes exposed to NBCCS fibroblasts in organotypic invasion assays. To do so, NBCCS primary keratinocytes were transduced using retroviral vectors allowing expression of the HPV16 E6-E7 protein, and hence the ubiquitination and proteasome degradation of P53 (Fig 7A). Under these circumstances, both WT E6-E7 and NBCCS E6-E7 keratinocytes became invasive in the presence of NBCCS fibroblasts (Fig 7B and 7C, S4 Fig). Since expression of the HPV16 E6 and E7 oncoproteins not only abrogates expression of P53 but also of the Retinoblastoma 1 protein (Rb1), we also introduced human keratinocytes isolated from human squamous cell carcinoma as a control (SCC13 [36]). Alike WT keratinocytes transformed by the E6 E7 oncoproteins, SCC13 cells became highly invasive in organotypic skin cultures containing NBCSS but not WT fibroblasts (not shown). These observations argue in favor of a central role of P53 in counteracting alterations of the dermo epidermal dialog led in the presence of NBCCS fibroblasts.

## Discussion

BCCs are skin cancers that exceptionally metastasize but may present with a high potential of local invasiveness. Since the early 60^s^, [37, 38] it has been hypothesized that growth of BCC cells depends on specific micro environmental conditions. This notion is further supported by the fact that in contrast to SCC cells, [36] BCC cells cannot be propagated *ex vivo*. Here, we present experimental evidences showing that dermal fibroblasts isolated from non-involved non photo-exposed skin biopsies from NBCCS patients severely impact behavior of WT keratinocytes in OSC. Most importantly, our data indicated that the presence of primary NBCCS dermal fibroblasts in dermis equivalents resulted in P53 stabilization and cell cycle perturbations, a situation reminiscent of keratinocytes response toward genotoxic stress.

In a previous study we showed that primary NBCCS fibroblasts expressed *ex vivo* and, in a cell autonomous manner (i.e. in absence of keratinocytes), a transcriptional signature of 18 genes [17] closely similar to that reported in fibroblasts associated to human BCCs [18]. Importantly, a portion of this signature was conserved irrespective of the 2D or 3D culture conditions, suggesting that *PATCHED1* heterozygosity constitutively impacts gene expression in NBCCS fibroblasts. The transcriptional signature of NBCCS fibroblasts included genes associated with tumor cell invasion, metastasis, and neoangiogenesis. Notably, NBCCS fibroblasts expressed higher mRNA levels of the anti-angiogenic protein ANGPTL4 which is known to limit tumor cell motility and invasiveness [39] and the invasive potential of BCC cells, as well [40, 41]. Also, we noticed an important decrease of *COL7A1* mRNA in NBCCS primary fibroblasts. Deleterious mutations of *COL7A1* have been associated with the development of aggressive SCCs in patients suffering from the recessive Hallopeau-Siemens dystrophic epidermolysis syndrome (HS-RDEB) [42] while its restoration delayed tumor cell dissemination *in vitro* and *in vivo* [43]. In HS-RDEB fibroblasts, abrogation of COL7A1 expression has also been shown associated with increased levels of *WNT5A* mRNAs [43]. *WNT5A* expression was also found augmented *in vivo*, in the stroma from pancreas cancers [31] and in fibroblasts surrounding both cutaneous SCC and BCC cells [44]. In spite of these observations, NBCCS fibroblasts exhibited decreased levels of *WNT5A* mRNAs, while, paradoxically, some markers of the activation of the WNT pathway (*WISP2* and *ID2* mRNAs) where found increased. The mechanisms by which heterozygosity of *PATCHED1* in NBCCS primary fibroblasts orchestrate the interplay between the SHH and WNT pathways then deserves further research.

Experiments in transgenic mice have shown that development and maintenance of BCC-like lesions depend on either ectopic and sustained secretion of SHH in the basal germinative epidermal keratinocytes, [45, 46] or from overexpression of GLI1 [47] or of GLI2 [48]. Our results in human cells indicated that *SHH* mRNAs were undetectable in keratinocytes and barely detectable in fibroblasts cultures. Surprisingly enough, and for the first time, cultures of WT keratinocytes with WT fibroblasts resulted in detectable SHH secretion. The amount of secreted SHH was further increased in co-cultures of WT keratinocytes and NBCCS fibroblasts. These results strongly suggested that NBCCS fibroblasts play a key role in the regulation of the SHH/PATCHED pathway in epidermal keratinocytes. In good agreement with these results, we showed that expression of endogenous *PATCHED1* mRNA and the activity of the 5’ regulatory region of the *PATCHED1* gene were slightly but reproducibly increased in WT keratinocytes maintained in the presence of culture supernatants conditioned by NBCCS fibroblasts. Ectopic expression of SHH in murine basal keratinocytes has been suggested to result in increased proliferation and tumor development [11]. Together with clinical observations showing that NBCCS BBCs may occur in non photo-exposed skin areas, these observations suggest that NBCCS fibroblasts could play a decisive role in the onset of BCCs. It is very important to notice that, due to the failure to grow BCC cells in culture, we could not assess the impact of NBCCS fibroblasts on BCC cells behavior under *in vivo* settings, i.e. using mouse as a vector of BCCs development.

In addition to these observations, OSC composed of NBCCS fibroblasts and WT keratinocytes exhibited abnormal traits of epidermal stratification and differentiation with thinning of the epidermal compartment. The presence of NBCCS fibroblasts also resulted in the formation of dermo-epidermal clefts, a feature commonly found at the periphery of human BCC *in vivo*, [28, 49] but not described in non-lesional skin from NBCCS patients [5]. It is conceivable that dermo-epidermal clefts found in OSC were to be connected to our observations showing accumulation of MMP1 and MMP3 mRNAs in NBCCS primary fibroblasts [17].

In addition to epidermal thinning and dermo-epidermal clefting, severe alterations of the stepwise program of epidermal differentiation were noticed. β1 Integrin was extended to the suprabasal epidermal layers, where it is generally low or undetectable, while K10 Keratin was found delayed (K10) and Loricrin severely decreased (Loricrin) in the presence of NBCCS fibroblasts. Morphological defects and terminal differentiation were then partly normalized in the presence of the SHH-blocking antibody 5E1, further supporting the idea that over secretion of SHH by NBCCS dermal fibroblasts in the presence of WT keratinocytes may affect epidermal homeostasis. Traits of delayed differentiation in sporadic skin tumors or in psoriasis skin lesions [50] are usually associated with hyper proliferation. Unexpectedly however, in spite of delayed epidermal differentiation, we observed a sharp decrease of Ki67-positive keratinocytes overlaying NBCCS fibroblasts, indicating that NBCCS fibroblasts alter proliferation of WT keratinocytes. Together with decreased numbers of Ki67-positive cells, the P53 protein was found stabilized in epidermal cells either overlaying a NBCCS dermis equivalent or following incubation in culture supernatants of NBCCS fibroblasts. In spite of P53 stabilization, no sign of apoptosis was detected in sections of OSC containing NBCCS fibroblasts (not shown). In addition, in 2D culture conditions, we failed to detect any increase in the stabilisation of P21 which is a direct transcriptional target of P53. These observations suggest that although they promote P53 stabilization in WT keratinocytes, culture supernatants of NBCCS fibroblasts are not able to activate the P21 checkpoint in 2D culture conditions, and/or in the absence of a fibroblast-keratinocyte dialog, as observed for N-SHH (over)expression (in NBCCS fibroblasts). Since stabilization of P53 results from exposure to genotoxic stresses [29] including ROS, [51, 52] we hypothesized that NBCCS fibroblasts could be responsible for such a stress in keratinocytes. Under our experimental settings, no ROS accumulation could be detected in WT keratinocytes treated with culture supernatants conditioned by NBCCS fibroblasts. However, in the frame of the same experimental design, WT keratinocytes cultured in NBCCS fibroblasts conditioned medium exhibited substantial increase of SA-β-Gal activity. In spite of this, neither the P16 nor the P21 markers of senescence [53] were stabilized under our experimental conditions, but rather appeared in reduced amounts. Further analyses should indicate whether NBCCS fibroblasts provoke an atypical mechanism of senescence irrespective of 2D or 3D cell culture conditions, or, alternatively, that triggering of a *bona fide* senescent mechanism depends on the presence of keratinocytes (in 3D organotypic skin cultures).

The mechanism by which secretory components from NBCCS fibroblasts can affect expression of gatekeeper proteins such as P53 in keratinocytes deserves further investigations. Nevertheless, we observed that strong attenuation of P53 expression in keratinocytes immortalized by the oncogenic HPV-16 E6-E7 proteins elicited epidermal invasions in the presence of NBCCS fibroblasts. Altogether, these observations suggested that fibroblasts-led stabilization of P53 in keratinocytes ensues from a yet unidentified genotoxic stress. In NBCCS patients, dermal fibroblasts could thus further promote the development of numerous BCCs before or after abrogation of P53 following an environmental mutagenic stress, as reported in more 50% skin cancers.

In conclusion, our observations further document the role of the SHH/PATCHED pathway in cutaneous homeostasis in the human. We propose that susceptibility of NBCCS patients towards BCCs could be partly due to a “pre activated state” of stromal fibroblasts. Normalizing fibroblasts “pre activation” using appropriate pharmacological substances could improve the high susceptibility of NBCCS patients toward BCCs development and, presumably, of human cancers associated to unbalanced SHH/PATCHED pathway.

## Supporting Information

S1 Fig Alteration of early markers of epidermal differentiation in NBCCS skins *in vivo*. Paraffin sections of non photo-exposed skin from WT (WT1), NBCCS6 and NBCCS10 patients were subjected to immunolabelling for Laminin B1 and β1 Integrin, as indicated. Note the increased deposition of both β1 Integrin and Laminin B1 as observed in OSC containing NBCCS fibroblasts. Bar: 100 μm.
(TIF)

S2 Fig Effects of NBCCS culture supernatant on ROS production in WT keratinocytes. The accumulation of ROS in WT keratinocytes was measured using the fluorescent dye DCFH-DA and FACS analysis. Upper panels, WT keratinocytes without treatment (Control). WT keratinocytes treated with non-conditioned fibroblast medium (NCM) or non-conditioned fibroblast medium + H202 (NCM + H202) as positive control of induced-ROS production. Lower panels, WT keratinocytes treated with culture media conditioned by either WT (WT1) or NBCCS (NBCCS6, NBCCS10) fibroblasts. Pre-treatment of WT keratinocytes with NBCCS culture supernatants did not led to detectable increase of ROS production.
(TIF)

S3 Fig Effect of NBCCS culture supernatants on P21 and P16 proteins expression in WT and WT E6-E7 keratinocytes. Western blot analysis showed decreased expression of P16 and P21 in WT keratinocytes treated with culture supernatants conditioned by NBCCS fibroblasts. P16 was also decreased in WT1 E6-E7 cells. P21 was not expressed in WT1 E6-E7 keratinocytes. Right panels show levels of P21 and P16 relative to Tubulin and expressed as fold induction relative to the WT fibroblast strain.
(TIF)

S4 Fig Full sections organotypic invasion assays. Images were taken all along the length of the organotypic sections and assembled using Image Composite Editor (ICE) software.
(TIF)

S1 Table Patients and cells characteristics. (DOCX)

S2 Table Specific TaqMan^®^ primers used for quantitative real-time PCR analysis. (DOC)

## References

[1] Sarasin A. The molecular pathways of ultraviolet-induced carcinogenesis. Mutat Res. 1999;428(1–2):5–10. 10517972 10.1016/s1383-5742(99)00025-3

[2] DePinho RA. The age of cancer. Nature. 2000;408(6809):248–54. 11089982 10.1038/35041694

[3] Gorlin R. Nevoid basal-cell carcinoma syndrome. Medicine (Baltimore). 1987;66(2):98–113. 3547011 10.1097/00005792-198703000-00002

[4] Shanley S, Ratcliffe J, Hockey A, Haan E, Oley C, Ravine D, et al Nevoid basal cell carcinoma syndrome: review of 118 affected individuals. Am J Med Genet. 1994;50(3):282–90. 8042673 10.1002/ajmg.1320500312

[5] Pruvost-Balland C, Gorry P, Boutet N, Magnaldo T, Mamelle G, Margulis A, et al [Clinical and genetic study in 22 patients with basal cell nevus syndrome]. Ann Dermatol Venereol. 2006;133(2):117–23. 16508594 10.1016/s0151-9638(06)70861-4

[6] Goldstein AM, Bale SJ, Peck GL, DiGiovanna JJ. Sun exposure and basal cell carcinomas in the nevoid basal cell carcinoma syndrome. J Am Acad Dermatol. 1993;29(1):34–41. 8315076 10.1016/0190-9622(93)70148-m

[7] Cleaver JE, Lam ET, Revet I. Disorders of nucleotide excision repair: the genetic and molecular basis of heterogeneity. Nat Rev Genet. 2009;10(11):756–68. Epub 2009/10/08. 10.1038/nrg2663 19809470

[8] Marigo V, Davey RA, Zuo Y, Cunningham JM, Tabin CJ. Biochemical evidence that patched is the Hedgehog receptor. Nature. 1996;384(6605):176–9. 8906794 10.1038/384176a0

[9] Stone DM, Hynes M, Armanini M, Swanson TA, Gu Q, Johnson RL, et al The tumour-suppressor gene patched encodes a candidate receptor for Sonic hedgehog [see comments]. Nature. 1996;384(6605):129–34. 8906787 10.1038/384129a0

[10] Lindstrom E, Shimokawa T, Toftgard R, Zaphiropoulos PG. PTCH mutations: distribution and analyses. Hum Mutat. 2006;27(3):215–9. 16419085 10.1002/humu.20296

[11] Scales SJ, de Sauvage FJ. Mechanisms of Hedgehog pathway activation in cancer and implications for therapy. Trends Pharmacol Sci. 2009;30(6):303–12. Epub 2009/05/16. 10.1016/j.tips.2009.03.007 19443052

[12] Soufir N, Gerard B, Portela M, Brice A, Liboutet M, Saiag P, et al PTCH mutations and deletions in patients with typical nevoid basal cell carcinoma syndrome and in patients with a suspected genetic predisposition to basal cell carcinoma: a French study. Br J Cancer. 2006;95(4):548–53. Epub 2006/08/16. 16909134 10.1038/sj.bjc.6603303 PMC2360669

[13] Bonifas JM, Bare JW, Kerschmann RL, Master SP, Epstein EH Jr. Parental origin of chromosome 9q22.3-q31 lost in basal cell carcinomas from basal cell nevus syndrome patients. Hum Mol Genet. 1994;3(3):447–8. 8012356 10.1093/hmg/3.3.447

[14] Kalluri R, Zeisberg M. Fibroblasts in cancer. Nat Rev Cancer. 2006;6(5):392–401. Epub 2006/03/31. 16572188 10.1038/nrc1877

[15] Radisky DC, Levy DD, Littlepage LE, Liu H, Nelson CM, Fata JE, et al Rac1b and reactive oxygen species mediate MMP-3-induced EMT and genomic instability. Nature. 2005;436(7047):123–7. 16001073 10.1038/nature03688 PMC2784913

[16] Brellier F, Valin A, Chevallier-Lagente O, Gorry P, Avril MF, Magnaldo T. Ultraviolet responses of Gorlin syndrome primary skin cells. Br J Dermatol. 2008;159(2):445–52. Epub 2008/05/31. 10.1111/j.1365-2133.2008.08650.x 18510667

[17] Valin A, Barnay-Verdier S, Robert T, Ripoche H, Brellier F, Chevallier-Lagente O, et al PTCH1 +/- dermal fibroblasts isolated from healthy skin of Gorlin syndrome patients exhibit features of carcinoma associated fibroblasts. PLoS One. 2009;4(3):e4818 Epub 2009/03/17. 10.1371/journal.pone.0004818 19287498 PMC2654107

[18] Micke P, Kappert K, Ohshima M, Sundquist C, Scheidl S, Lindahl P, et al In situ identification of genes regulated specifically in fibroblasts of human basal cell carcinoma. J Invest Dermatol. 2007;127(6):1516–23. 17273163 10.1038/sj.jid.5700714

[19] Bissell MJ, Hines WC. Why don't we get more cancer? A proposed role of the microenvironment in restraining cancer progression. Nat Med. 2011;17(3):320–9. 10.1038/nm.2328 21383745 PMC3569482

[20] Rheinwald JG, Green H. Epidermal growth factor and the multiplication of cultured human epidermal keratinocytes. Nature. 1977;265(5593):421–4. Epub 1977/02/03. 299924 10.1038/265421a0

[21] Halbert CL, Demers GW, Galloway DA. The E7 gene of human papillomavirus type 16 is sufficient for immortalization of human epithelial cells. J Virol. 1991;65(1):473–8. Epub 1991/01/01. 1845902 10.1128/jvi.65.1.473-478.1991 PMC240541

[22] Asselineau D, Bernard B, Bailly C, Darmon M. Epidermal morphogenesis and induction of the 67 kD keratin polypeptide by culture of human keratinocytes at the liquid-air interface. Exp Cell Res. 1985;159(2):536–9. 2411581 10.1016/s0014-4827(85)80027-6

[23] Gaggioli C, Hooper S, Hidalgo-Carcedo C, Grosse R, Marshall JF, Harrington K, et al Fibroblast-led collective invasion of carcinoma cells with differing roles for RhoGTPases in leading and following cells. Nat Cell Biol. 2007;9(12):1392–400. Epub 2007/11/27. 18037882 10.1038/ncb1658

[24] Brellier F, Bergoglio V, Valin A, Barnay S, Chevallier-Lagente O, Vielh P, et al Heterozygous mutations in the tumor suppressor gene PATCHED provoke basal cell carcinoma-like features in human organotypic skin cultures. Oncogene. 2008;27(51):6601–6. Epub 2008/08/06. 10.1038/onc.2008.260 18679421

[25] Magnaldo T, Bernerd F, Asselineau D, Darmon M. Expression of loricrin is negatively controlled by retinoic acid in human epidermis reconstructed in vitro. Differentiation. 1992;49(1):39–46. 1378029 10.1111/j.1432-0436.1992.tb00767.x

[26] Traiffort E, Moya KL, Faure H, Hassig R, Ruat M. High expression and anterograde axonal transport of aminoterminal sonic hedgehog in the adult hamster brain. Eur J Neurosci. 2001;14(5):839–50. 11576188 10.1046/j.0953-816x.2001.01708.x

[27] Brellier F, Marionnet C, Chevallier-Lagente O, Toftgard R, Mauviel A, Sarasin A, et al Ultraviolet irradiation represses PATCHED gene transcription in human epidermal keratinocytes through an activator protein-1-dependent process. Cancer Res. 2004;64(8):2699–704. 15087382 10.1158/0008-5472.can-03-3477

[28] Ulrich M, Roewert-Huber J, Gonzalez S, Rius-Diaz F, Stockfleth E, Kanitakis J. Peritumoral clefting in basal cell carcinoma: correlation of in vivo reflectance confocal microscopy and routine histology. J Cutan Pathol. 2011;38(2):190–5. 10.1111/j.1600-0560.2010.01632.x 21039746

[29] Lane D, Levine A. p53 Research: the past thirty years and the next thirty years. Cold Spring Harb Perspect Biol. 2010;2(12):a000893 10.1101/cshperspect.a000893 20463001 PMC2982174

[30] Bhowmick NA, Chytil A, Plieth D, Gorska AE, Dumont N, Shappell S, et al TGF-beta signaling in fibroblasts modulates the oncogenic potential of adjacent epithelia. Science. 2004;303(5659):848–51. Epub 2004/02/07. 14764882 10.1126/science.1090922

[31] Pilarsky C, Ammerpohl O, Sipos B, Dahl E, Hartmann A, Wellmann A, et al Activation of Wnt signalling in stroma from pancreatic cancer identified by gene expression profiling. J Cell Mol Med. 2008;12(6B):2823–35. 10.1111/j.1582-4934.2008.00289.x 18298655 PMC3828895

[32] Zhu P, Tan MJ, Huang RL, Tan CK, Chong HC, Pal M, et al Angiopoietin-like 4 protein elevates the prosurvival intracellular O2(-):H2O2 ratio and confers anoikis resistance to tumors. Cancer Cell. 2011;19(3):401–15. 10.1016/j.ccr.2011.01.018 21397862

[33] Campisi J, Yaswen P. Aging and cancer cell biology, 2009. Aging Cell. 2009;8(3):221–5. 10.1111/j.1474-9726.2009.00475.x 19627264

[34] Zuniga A, Zeller R. Development. In Turing's hands—the making of digits. Science. 2014;345(6196):516–7. 10.1126/science.1257501 25082687

[35] Petitjean A, Mathe E, Kato S, Ishioka C, Tavtigian SV, Hainaut P, et al Impact of mutant p53 functional properties on TP53 mutation patterns and tumor phenotype: lessons from recent developments in the IARC TP53 database. Hum Mutat. 2007;28(6):622–9. 17311302 10.1002/humu.20495

[36] Rheinwald JG, Beckett MA. Tumorigenic keratinocyte lines requiring anchorage and fibroblast support cultured from human squamous cell carcinomas. Cancer Res. 1981;41(5):1657–63. 7214336

[37] Van Scott EJ, Reinertson RP. The modulating influence of stromal environment on epithelial cells studied in human autotransplants. J Invest Dermatol. 1961;36:109–31. 13780053

[38] Lyles TW, Freeman RG, Knox JM. Transplantation of basel cell epitheliomas. J Invest Dermatol. 1960;34:353 14419128

[39] Zhang Y, Hu X, Tian R, Wei W, Hu W, Chen X, et al Angiopoietin-related growth factor (AGF) supports adhesion, spreading, and migration of keratinocytes, fibroblasts, and endothelial cells through interaction with RGD-binding integrins. Biochem Biophys Res Commun. 2006;347(1):100–8. 16806062 10.1016/j.bbrc.2006.06.053

[40] Galaup A, Cazes A, Le Jan S, Philippe J, Connault E, Le Coz E, et al Angiopoietin-like 4 prevents metastasis through inhibition of vascular permeability and tumor cell motility and invasiveness. Proc Natl Acad Sci U S A. 2006;103(49):18721–6. 17130448 10.1073/pnas.0609025103 PMC1693729

[41] Tan MJ, Teo Z, Sng MK, Zhu P, Tan NS. Emerging roles of angiopoietin-like 4 in human cancer. Mol Cancer Res. 2012;10(6):677–88. 10.1158/1541-7786.MCR-11-0519 22661548

[42] Fine JD, Johnson LB, Weiner M, Li KP, Suchindran C. Epidermolysis bullosa and the risk of life-threatening cancers: the National EB Registry experience, 1986–2006. J Am Acad Dermatol. 2009;60(2):203–11. 10.1016/j.jaad.2008.09.035 19026465

[43] Ng YZ, Pourreyron C, Salas-Alanis JC, Dayal JH, Cepeda-Valdes R, Yan W, et al Fibroblast-derived dermal matrix drives development of aggressive cutaneous squamous cell carcinoma in patients with recessive dystrophic epidermolysis bullosa. Cancer Res. 2012;72(14):3522–34. 10.1158/0008-5472.CAN-11-2996 22564523

[44] Pourreyron C, Reilly L, Proby C, Panteleyev A, Fleming C, McLean K, et al Wnt5a is strongly expressed at the leading edge in non-melanoma skin cancer, forming active gradients, while canonical Wnt signalling is repressed. PLoS One. 2012;7(2):e31827 10.1371/journal.pone.0031827 22384081 PMC3285195

[45] Oro AE, Higgins KM, Hu Z, Bonifas JM, Epstein EH, Jr., Scott MP. Basal cell carcinomas in mice overexpressing sonic hedgehog. Science. 1997;276(5313):817–21. 9115210 10.1126/science.276.5313.817

[46] Adolphe C, Narang M, Ellis T, Wicking C, Kaur P, Wainwright B. An in vivo comparative study of sonic, desert and Indian hedgehog reveals that hedgehog pathway activity regulates epidermal stem cell homeostasis. Development. 2004;131(20):5009–19. 15371305 10.1242/dev.01367

[47] Nilsson M, Unden AB, Krause D, Malmqwist U, Raza K, Zaphiropoulos PG, et al Induction of basal cell carcinomas and trichoepitheliomas in mice overexpressing GLI-1. Proc Natl Acad Sci U S A. 2000;97(7):3438–43. 10725363 10.1073/pnas.050467397 PMC16258

[48] Grachtchouk M, Mo R, Yu S, Zhang X, Sasaki H, Hui CC, et al Basal cell carcinomas in mice overexpressing Gli2 in skin. Nat Genet. 2000;24(3):216–7. Epub 2000/03/04. 10700170 10.1038/73417

[49] Boone MA, Norrenberg S, Jemec GB, Del Marmol V. Imaging of basal cell carcinoma by high-definition optical coherence tomography: histomorphological correlation. A pilot study. Br J Dermatol. 2012;167(4):856–64. 10.1111/j.1365-2133.2012.11194.x 22862425

[50] Bernerd F, Magnaldo T, Darmon M. Delayed onset of epidermal differentiation in psoriasis. J Invest Dermatol. 1992;98(6):902–10. 1375620 10.1111/1523-1747.ep12460344

[51] de Gruijl FR. Photocarcinogenesis: UVA vs. UVB radiation. Skin Pharmacol Appl Skin Physiol. 2002;15(5):316–20. 12239425 10.1159/000064535

[52] Nishigori C, Hattori Y, Toyokuni S. Role of reactive oxygen species in skin carcinogenesis. Antioxid Redox Signal. 2004;6(3):561–70. 15130282 10.1089/152308604773934314

[53] Kim RH, Kang MK, Kim T, Yang P, Bae S, Williams DW, et al Regulation of p53 during senescence in normal human keratinocytes. Aging Cell. 2015;14(5):838–46. 10.1111/acel.12364 26138448 PMC4568971

