# Supplementary figures and images for: Basal Cell Carcinoma in Gorlin’s Patients: a Matter of Fibroblasts-Led Protumoral Microenvironment?
Source: PLoS One. 2015 Dec 22;10(12):e0145369. doi: 10.1371/journal.pone.0145369 (PMC4687848; doi:10.1371/journal.pone.0145369)

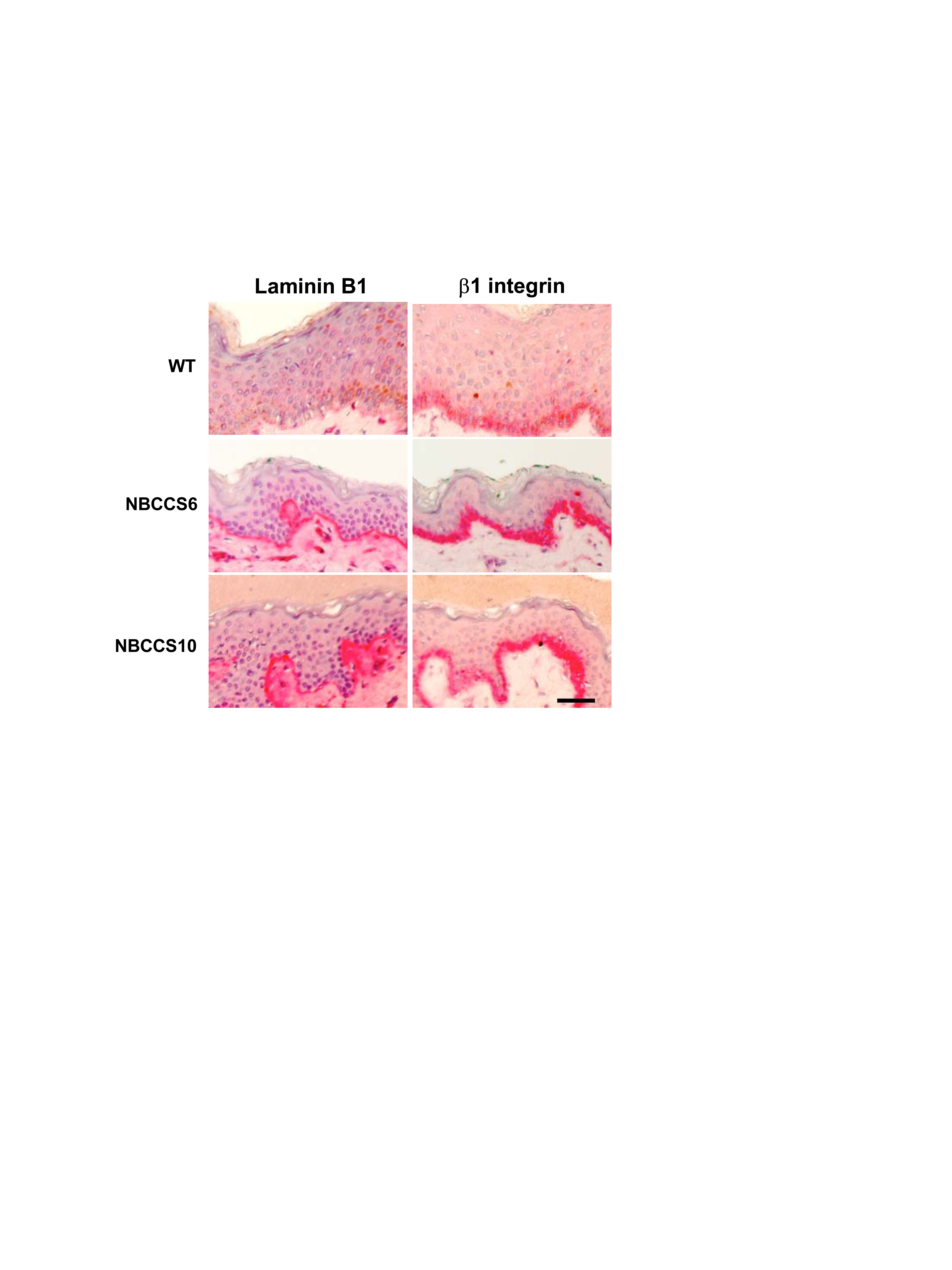

Supplement: S1 Fig — Paraffin sections of non photo-exposed skin from WT (WT1), NBCCS6 and NBCCS10 patients were subjected to immunolabelling for Laminin B1 and β1 Integrin, as indicated. Note the increased deposition of both β1 Integrin and Laminin B1 as observed in OSC containing NBCCS fibroblasts. Bar: 100 μm. (TIF) [file pone.0145369.s001.tif]

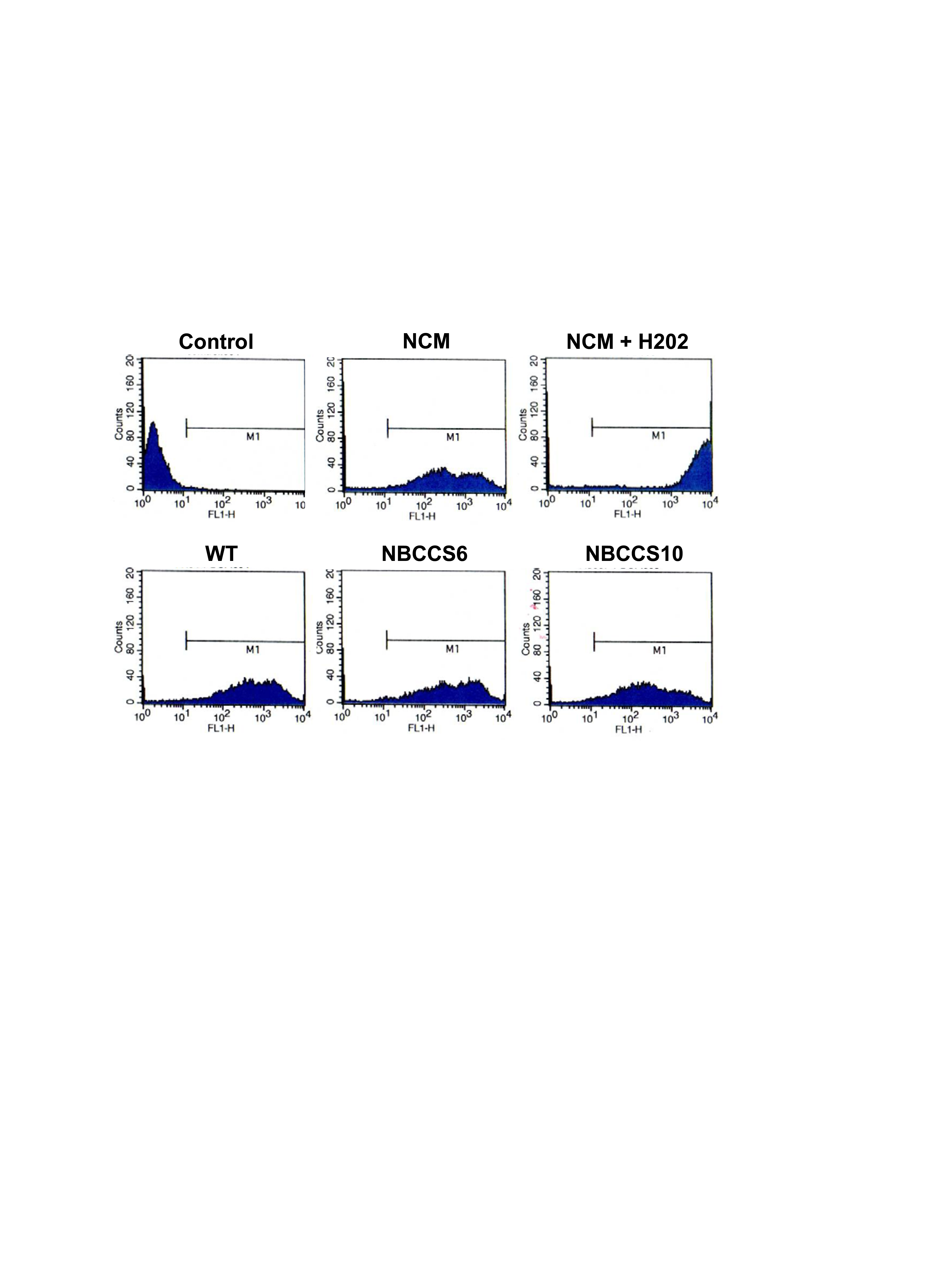

Supplement: S2 Fig — The accumulation of ROS in WT keratinocytes was measured using the fluorescent dye DCFH-DA and FACS analysis. Upper panels, WT keratinocytes without treatment (Control). WT keratinocytes treated with non-conditioned fibroblast medium (NCM) or non-conditioned fibroblast medium + H202 (NCM + H202) as positive control of induced-ROS production. Lower panels, WT keratinocytes treated with culture media conditioned by either WT (WT1) or NBCCS (NBCCS6, NBCCS10) fibroblasts. Pre-treatment of WT keratinocytes with NBCCS culture supernatants did not led to detectable increase of ROS production. (TIF) [file pone.0145369.s002.tif]

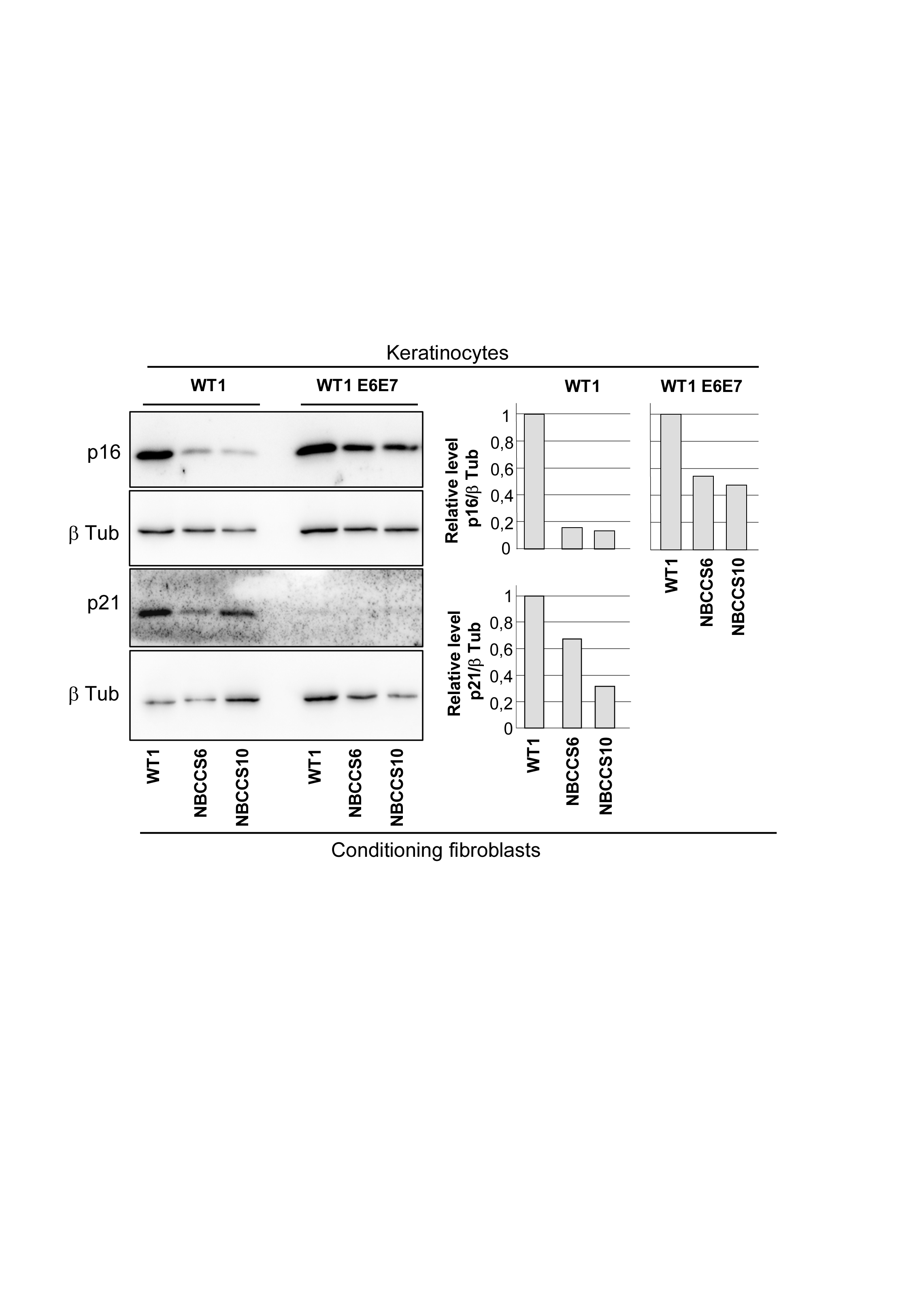

Supplement: S3 Fig — Western blot analysis showed decreased expression of P16 and P21 in WT keratinocytes treated with culture supernatants conditioned by NBCCS fibroblasts. P16 was also decreased in WT1 E6-E7 cells. P21 was not expressed in WT1 E6-E7 keratinocytes. Right panels show levels of P21 and P16 relative to Tubulin and expressed as fold induction relative to the WT fibroblast strain. (TIF) [file pone.0145369.s003.tif]

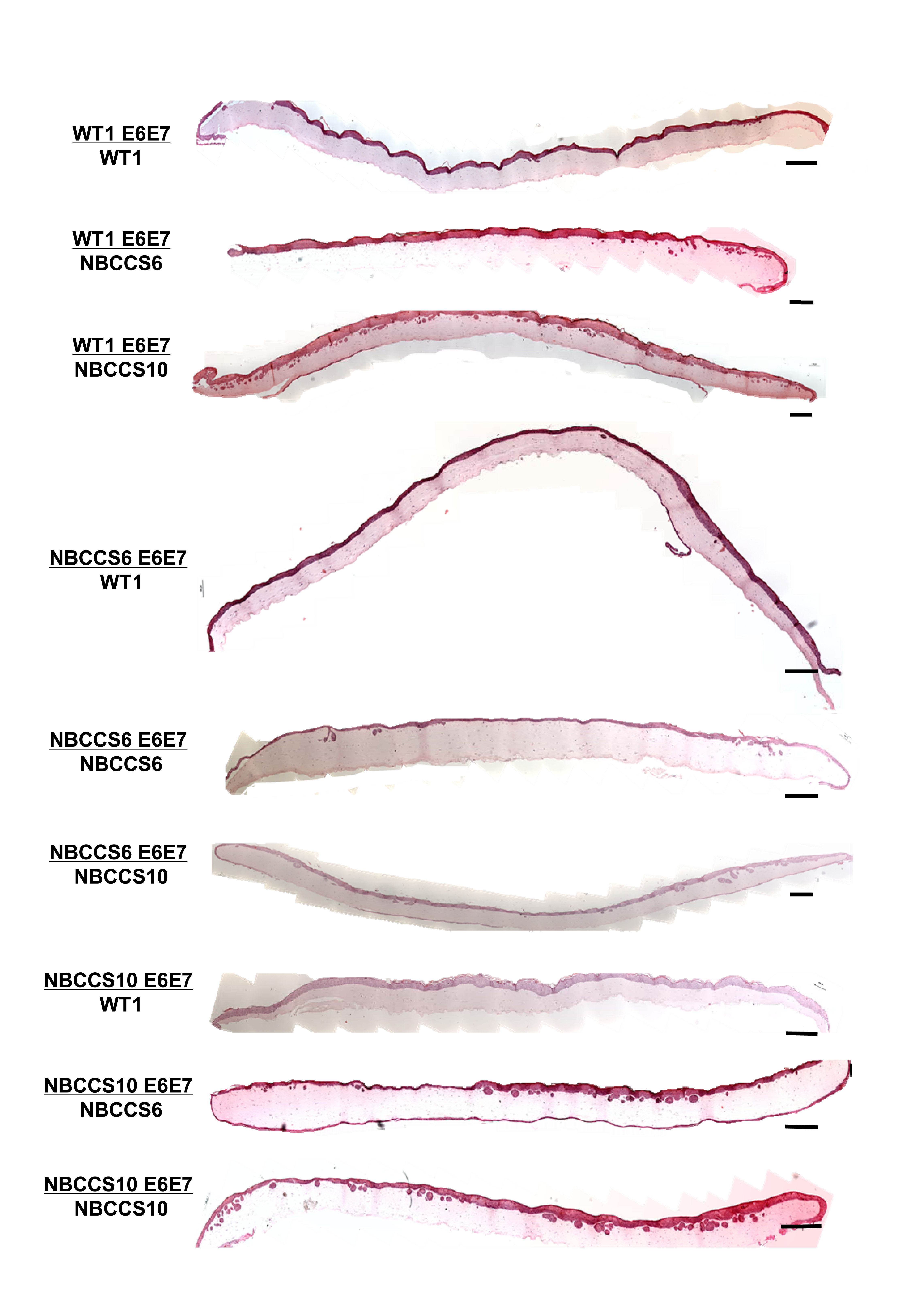

Supplement: S4 Fig — Images were taken all along the length of the organotypic sections and assembled using Image Composite Editor (ICE) software. (TIF) [file pone.0145369.s004.tif]
