# Supplementary material for: Basal Cell Carcinoma in Gorlin’s Patients: a Matter of Fibroblasts-Led Protumoral Microenvironment?
Source: PLoS One. 2015 Dec 22;10(12):e0145369. doi: 10.1371/journal.pone.0145369 (PMC4687848; doi:10.1371/journal.pone.0145369)
Supplement: S1 Table — (DOCX) [file pone.0145369.s005.docx]

Table S1 : Patients and cells characteristics.

| Diagnosis | Patients | Age at Biopsy | Ethnic origin | *PATCHED1* mutation |
| --- | --- | --- | --- | --- |
| CTRL | WT1 | 30 | caucasian | NA |
|  | WT2 | 16 | caucasian | NA |
|  | WT3 | 28 | black | NA |
| NBCCS | NBCCS1 | 78 | caucasian | c.3422C>T ; p.Ala1141Val |
|  | NBCCS7 | 78 | caucasian | c.3583A>T ; p.Thr1195Ser |
|  | NBCCS8 | 28 | caucasian | c.2199A>G ; p.Ser733Ser |
|  | NBCCS3 | 42 | caucasian | c.1762supG ; p.Val588GlyfsX39 |
|  | NBCCS6 | 45 | caucasian | c.1925dipC ; p.Pro643ThrfsX11 |
|  | NBCCS10 | 43 | caucasian | c.1366del ; p.Thr456ProfsX35 |

CTRL : Controls. NBCCS : Nevoid Basal Cell Carcinoma Syndrome. WT : wild type. NA : not applicable
