# Supplementary material for: Basal Cell Carcinoma in Gorlin’s Patients: a Matter of Fibroblasts-Led Protumoral Microenvironment?
Source: PLoS One. 2015 Dec 22;10(12):e0145369. doi: 10.1371/journal.pone.0145369 (PMC4687848; doi:10.1371/journal.pone.0145369)
Supplement: S2 Table — (DOC) [file pone.0145369.s006.doc]

Table S2 : Primers used for quantitative real-time PCR

| **Gene Name** | **TaqMan® Gene Expression Assay Reference** |
| --- | --- |
| *GAPDH* | Hs99999905_m1 |
| *PPIA* | Hs99999904_m1 |
| *RPLO1* | Hs99999902_m1 |
| *B2M* | Hs99999907_m1 |
| *TBP* | Hs99999910_m1 |
| *MMP1* | Hs00233958_m1 |
| *MMP3* | Hs00233962_m1 |
| *COL3A1* | Hs00943809_m1 |
| *COL7A1* | Hs00164310_m1 |
| *COL11A1* | Hs00266273_m1 |
| *LAMA2* | Hs00166308_m1 |
| *TNC* | Hs01115664_m1 |
| *CXCL12* | Hs00171022_m1 |
| *MGP* | Hs00179899_m1 |
| *ANGPTL2* | Hs00765775_m1 |
| *ANGPTL4* | Hs00211522_m1 |
| *FGF7* | Hs00940253_m1 |
| *GREM1* | Hs00171951_m1 |
| *SFRP2* | Hs00293258_m1 |
| *DKK3* | Hs00247426_m1 |
| *WNT5A* | Hs00180103_m1 |
| *WISP2* | Hs00180242_m1 |
| *ID2* | Hs00747379_m1 |
